# How Could the Use of Crop Wild Relatives in Breeding Increase the Adaptation of Crops to Marginal Environments?

**DOI:** 10.3389/fpls.2022.886162

**Published:** 2022-06-16

**Authors:** Juan Pablo Renzi, Clarice J. Coyne, Jens Berger, Eric von Wettberg, Matthew Nelson, Soledad Ureta, Fernando Hernández, Petr Smýkal, Jan Brus

**Affiliations:** ^1^Instituto Nacional de Tecnología Agropecuaria, Hilario Ascasubi, Argentina; ^2^CERZOS, Departamento de Agronomía, Universidad Nacional del Sur (CONICET), Bahía Blanca, Argentina; ^3^USDA Agricultural Research Service, Pullman, WA, United States; ^4^Agriculture and Food, Commonwealth Scientific and Industrial Research Organisation, Wembley, WA, Australia; ^5^Department of Plant and Soil Science, Gund Institute for Environment, University of Vermont, Burlington, VT, United States; ^6^Department of Applied Mathematics, Peter the Great St. Petersburg Polytechnic University, Saint Petersburg, Russia; ^7^The UWA Institute of Agriculture, University of Western Australia, Crawley, WA, Australia; ^8^Department of Botany, Faculty of Science, Palacký University, Olomouc, Czechia; ^9^Department of Geoinformatics, Faculty of Sciences, Palacký University, Olomouc, Czechia

**Keywords:** abiotic stress, adaptation, breeding, crop wild relatives, legumes, marginal environment

## Abstract

Alongside the use of fertilizer and chemical control of weeds, pests, and diseases modern breeding has been very successful in generating cultivars that have increased agricultural production several fold in favorable environments. These typically homogeneous cultivars (either homozygous inbreds or hybrids derived from inbred parents) are bred under optimal field conditions and perform well when there is sufficient water and nutrients. However, such optimal conditions are rare globally; indeed, a large proportion of arable land could be considered marginal for agricultural production. Marginal agricultural land typically has poor fertility and/or shallow soil depth, is subject to soil erosion, and often occurs in semi-arid or saline environments. Moreover, these marginal environments are expected to expand with ongoing climate change and progressive degradation of soil and water resources globally. Crop wild relatives (CWRs), most often used in breeding as sources of biotic resistance, often also possess traits adapting them to marginal environments. Wild progenitors have been selected over the course of their evolutionary history to maintain their fitness under a diverse range of stresses. Conversely, modern breeding for broad adaptation has reduced genetic diversity and increased genetic vulnerability to biotic and abiotic challenges. There is potential to exploit genetic heterogeneity, as opposed to genetic uniformity, in breeding for the utilization of marginal lands. This review discusses the adaptive traits that could improve the performance of cultivars in marginal environments and breeding strategies to deploy them.

## Introduction

When coupled with the use of irrigation, fertilizers and pesticides, modern breeding has increased agricultural production several fold in favorable environments (Evenson and Gollin, 2003). However, such high-input agriculture also has a high environmental impact. High performing cultivars are designed under good field conditions and perform well when there is sufficient water and nutrients, e.g., fertile soil. However, such conditions are often not available because there is a large proportion of arable land in marginal environments globally (Sebby, 2010; Pingali, 2012). Marginal land exists worldwide (e.g., Kang et al., 2013), and typically, it is of poor soil fertility. In many areas these soils are prone to erosion, with shallow soil layers, and may be prone to drought, salinity, or other abiotic challenges. Moreover, these environments are expected to expand with ongoing climate change and increasing degradation of soil and water resources (El-Beltagy and Madkour, 2012).

Throughout evolutionary history, plants have had to adapt to a range of environmental conditions including adverse habitats. As a consequence, plant species including crop wild relatives typically exhibit a high level of genetic and phenotypic variation within their distribution range (Anderson et al., 2011; Russell et al., 2014; Smýkal et al., 2017; Hellwig et al., 2021). Understanding the genetic, biological and ecological bases of local adaptation is relevant to manage the impact of climate change on crop and animal production. In widely distributed plant species inhabiting different environments, ecotypic differentiation has been detected for populations along altitudinal, latitudinal or environmental gradients (e.g., Polechová and Barton, 2015; Berger et al., 2017). Understanding the extent to which such distinct ecotypes are formed will help not only to reveal and recognize their evolutionary adaptive patterns, but also will help breeders to use their diversity to maximize productivity under target environments.

It is widely accepted that past domestication processes have resulted in a narrower genetic base of cultivated germplasm that is prone to pests and diseases (Tanksley and McCouch, 1997; McCouch et al., 2020; Khoury et al., 2022). Unlike their domesticated crops, crop wild relatives (CWRs) continued to evolve to adapt to their environments and thus provide a key resource to counteract the effects of climate change on the world’s food supply. To fully understand and utilize this process, studies in the geographical centers of origin are needed combining ecology, physiology and genetics. This approach was pioneered by N.I. Vavilov in the 1920s–1930s starting with surveys, collecting missions and subsequent evaluation of germplasm collections in diverse environmental settings (Vavilov, 1957). Today, with the available tools of genomics, geospatial analysis and modeling combined with improved phenotyping methods, it has become all the more important to analyze wild accessions and use them as sources of stress tolerance for breeding purposes (Warschefsky et al., 2014; Coyne et al., 2020). Wild species have been used mainly for the introgression of disease and insect resistance into crops (e.g., Hajjar and Hodgkin, 2007); drought, cold, heat and salinity tolerance have also been addressed in some staple crops (Prohens et al., 2017). Biotic and abiotic stress resistances have been explored in wild relatives of many crops including chickpea, barley and maize (Brozynska et al., 2016; Fustier et al., 2017; von Wettberg et al., 2018; Liu et al., 2020). A complementary approach is the *de novo* domestication of new crops (Smýkal et al., 2018; Fernie and Yan, 2019).

Environmental fluctuations such as soil salinity, cold and drought stress represent a major constraint on agricultural productivity. Water availability and temperature extremes are the major factors controlling the distribution of vegetation over the earth’s surface. Crop yields are more dependent on an adequate supply of water than on any other single factor; environmental stress represents the primary cause of crop losses. With current climate change projections, extremely hot weather will become more frequent and rainfall will be more erratic in many regions of the world (Foyer et al., 2016). As many elite cultivars tend to be drought sensitive, ensuring food security will require development of more drought resistant varieties. Dissection of adaptation mechanisms to harsh conditions naturally occurring in wild relatives of domesticated crops may provide a solid foundation for crop improvement under the changing climate. Drought and heat stresses impact every plant developmental stage, starting from seed germination, through vegetative growth. However, the most adverse effects (from a crop production perspective) are on crop establishment (germination and young seedling phase) and reproduction, which are very sensitive to suboptimal conditions. Small seedlings and pollen grains happen to be particularly sensitive to lack of water and excess heat (Dürr et al., 2015; Yu et al., 2019; Chaturvedi et al., 2021; Chen et al., 2021).

## Marginal Environments and the Impact of Climate Change

The term “marginal” land originated in the field of agricultural economics during the 19th century (Kang et al., 2013) by Ricardo (2005) using the categorization from his land rent theory which was the basis of marginal productivity theory. Marginal lands are subject to socio-economic and biophysical constraints, and lower productivity. Nevertheless, it is difficult to define “productivity” within this concept because this varies depending on land use. For example, land that is too marginal for crop production might be suitable for grazing, and “fragile” land can be susceptible to soil degradation, but it may still be suitable for sustainable forestry. Many dry-land countries have not assessed the extent or characteristics of these lands, nor their sustainability as biofuels or food crops production. These lands are often difficult to exploit economically and sustainably for agriculture, and can lead to abandonment (Campbell et al., 2008). Land can also be degraded due to an intensive and unsustainable use (Campbell et al., 2008; Dauber et al., 2010). Marginal lands are areas with low rainfall, high temperatures, low quality soils, steep terrain, shallow soil depth (less than 50 cm), poor fertility, coarse texture, stony, heavy cracked clays, salt-affected lands, waterlogged, marshy land, barren and rocky soils, and other problems. If a land is considered marginal it means that it does not have enough capacity to produce food, or other agricultural activities.

**FIGURE 1:**
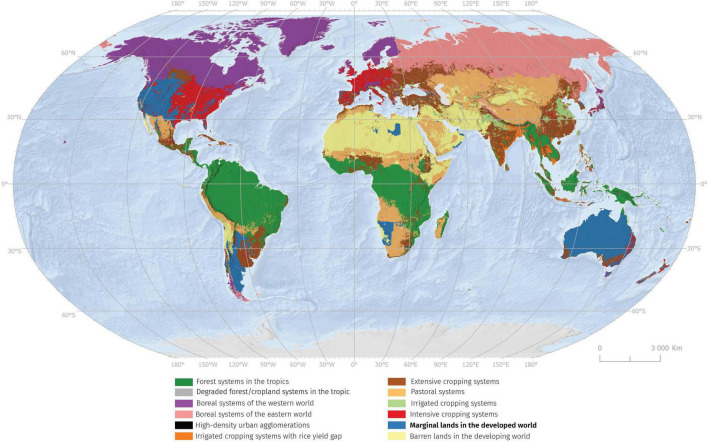
Map shows global land system archetypes classification by Václavík et al. (2013). Within the global scale, marginal lands in the developed world cover about 9% of terrestrial ecosystems.

Nearly one billion people live in poverty today, a high percentage living in these marginal lands in mostly developing countries (Ahmadzai et al., 2021). These areas with low soil fertility and limited agricultural value are also highly vulnerable to climate change (Castro and Castro, 2019). The research concluded that in total 29% of the agricultural area is marginal in European Union. The most common reason is the rooting limitations; 12% of Europe’s agricultural area required intervention to improve soil structure and depth to allow farming. This is followed by adverse climate and excessive soil moisture occurring in respectively 11 and 8% of the agricultural land (Elbersen et al., 2018). Within the global scale marginal lands in developed world covers about 9% of terrestrial ecosystems (Václavík et al., 2013). These lands occur predominantly in Western United States, Australia, Argentina, but also in North and South Africa (Figure 1). A more precise estimation of area covered by marginal lands is limited by uncertainties and problems of land cover classification (Brus et al., 2018). Marginal lands are most vulnerable to climate events like drought and floods, thus, most likely to be affected by climate change. Climate change has made it difficult to maintain established farming practices in recent decades. Within the US the prevalence of local yield deficits confirms that individual farmers looking to expand their operations are generally confined to cultivating increasingly marginal land, though widespread variation exists from location to location (Lark et al., 2020).

The effects of climate change are manifested as erratic precipitation and weather changes, which have led to rapid fluctuations and unpredictable temperatures. Adaptation to this requires new approaches to agriculture. For example, research on agro-climatic conditions has shown that farmers in dryland areas are more aware of climate change impacts than those in wetland areas. Similar findings have also been reported for Ecuador (Skarbø and VanderMolen, 2014). It is unclear, however, if agricultural producers can keep up with climate change at the incredible pace it is predicted to occur in coming years (Jones et al., 2012). Adaptation to climate change is difficult and impacts are based on various assumptions. In addition to estimating future impacts, researchers need to make subjective value judgments. This is especially true of social and economic development in marginal regions. As a result, the effects of climate change on these areas are difficult to forecast. Although the impacts of climate change are not always measurable, they are still a huge concern for governments and local communities alike. Some areas of the world are particularly vulnerable, and these risks can be mitigated by developing new approaches and technologies in crop production. One such approach is the implementation of local and regional adaptation plans. However, it is crucial to consider the broader context of marginal environments when developing policies and adaptation strategies for agricultural producers. This research is particularly important in Latin America, where smallholder farmers are heavily impacted by climate change. Moreover, rural and subsistence farming are often location-specific systems, which are highly vulnerable to the stressors of the climate. As a result, the research needs to take this into account. Agro-ecosystems are critical for food production and sustainability, but they are also prone to pests. Consequently, many current crop varieties will have to be replaced as a result of climate change. Failure to address these challenges will have a profound impact on the global economy and on social well-being. If we fail to protect and restore natural ecosystems, it will become impossible to grow crops in these marginal areas in the future. One of the possible way how to cope with this situation is implementation of Climate-Smart Agriculture (CSA) (Lipper et al., 2014).

## Context: How and Why Do Crops and Their Wild Relatives Differ?

Crop wild relatives typically harbor far greater genetic diversity than modern elite crops as a result of primary and secondary domestication bottlenecks; the founder effect(s) and subsequent fixation of domestication syndrome and post-domestication traits including cultural selection for local preference (Tanksley and McCouch, 1997; Purugganan and Fuller, 2009). Moreover, the evolutionary trajectory and resultant selection pressures encountered by wild progenitors and elite cultivars are very different. While we can now deploy an impressive array of technical and analytical approaches for handling complexity in the search for adaptive genes (Cortes and Lopez-Hernandez, 2021), here we argue that understanding the divergent selection pressures encountered by wild progenitor and elite cultivar adds invaluable context for the use of CWR in improving crop adaptation.

Wild progenitors are subject to natural selection in response to environmental stressors, herbivory, pathogen attack and inter-plant competition from the same or other species occupying similar ecological niches in a variable climate. While viable seed production is the ultimate measure of ‘fitness,’ reproductive investment in CWR is usually lower than in their domesticated counterparts (Berger et al., 2017; Berger J. et al., 2020; Garibaldi et al., 2021). This implies that competition for resources such as light, nutrients or water is selecting for greater vegetative investment (roots, shoots, leaves) in CWR relative to the domesticated crop, akin to the competitors in Grime’s triangle (Grime, 1974). In some examples the genomic consequences of this divergent selection for reproductive versus vegetative investment have been identified, such as the selection for increased apical dominance (e.g., reduced branching) in the domestication of maize (Clark et al., 2004). Seed dormancy, a key domestication syndrome trait, can both exacerbate and ameliorate seasonal variability in CWR. In many Mediterranean CWR seed physical dormancy has been selected as bet-hedging against false breaks (heavy rainfall that precedes the growing season start and which would expose the recently germinated seedling to extreme heat and drought) and other disturbances such as catastrophic herbivory (Piano et al., 1996; Norman et al., 2002; Smýkal et al., 2014; Berger et al., 2017). Typically, there is wide ranging dormancy within populations (Berger et al., 2017; Hradilová et al., 2019; Renzi et al., 2019), that in combination with variable seed placement will drive staggered germination across the CWR population over the growing season, exposing individuals to different micro-habitats in space and time.

Conversely, crop plants are subject to a combination of natural and artificial selection giving rise to the domestication syndrome (large seed size, loss of dormancy, and seed dehiscence etc.) and subsequent selection for local adaptation and food preferences (Fuller and Allaby, 2009; Purugganan and Fuller, 2009; Smýkal et al., 2018). Compared to CWR, modern crops lead a strictly regulated life in a tightly defined growing season. Non-dormant seeds are accurately positioned with respect to soil depth and neighbor distance in a prepared seedbed during an optimal window, matching phenology to target environment to minimize frost, drought, and other stress. Ripening is both uniform and timely because of the use of determinate cultivars (e.g., cereals), selection for earliness (e.g., chickpea, lupin, canola), or the application of desiccants (e.g., canola, chickpea). Herbivory and diseases are minimized by management interventions. Importantly, crops are selected on the basis of communal, rather than individual yield, whereas the opposite is the case for CWR. As a result, modern crops tend to be weak-moderate competitors (Donald, 1981; Weiner et al., 2017) with a greater reproductive investment than their wild progenitors (Berger et al., 2017; Berger J. et al., 2020; Garibaldi et al., 2021).

Finally, domestication and subsequent global dispersal further widens the evolutionary dichotomy between crop and CWR (Purugganan and Fuller, 2009). Chickpea represents an extreme exemplar, having been domesticated from a very narrowly distributed southeastern Anatolian wild progenitor with a Mediterranean winter annual lifecycle into a global crop grown on all continents except Antarctica. It has been hypothesized to have returned to the Mediterranean as a spring-sown late season crop after a 2000 years archeological gap (Abbo et al., 2003). The much more recently domesticated narrow-leafed lupin (*L. angustifolius*) and yellow lupin (*L. luteus*) represent the opposite extreme. Here the wild progenitors range widely around the Mediterranean basin (particularly *L. angustifolius*), being domesticated in the 18th century as spring-sown crops for limited sandy acid soil regions in central and eastern Europe (Hondelmann, 1984), and then returning to Mediterranean climates in the southern hemisphere in the last 50 years (Gladstones, 1994). The combination of divergent genetic diversity and evolutionary trajectory/selection pressures leads to different adaptive traits in crops and their wild relatives.

The regulation of phenology in Mediterranean crops and wild relatives provides clear examples of divergent evolutionary paths. For example, vernalization plays an important role in regulating phenology in both wild *Cicer* and *Lupinus*, presumably as a consequence of the cool-cold winters and rapidly warming spring temperatures experienced in their Mediterranean distribution range. Wild narrow-leafed and yellow lupins are widespread species in which flowering time and associated traits including water use, biomass production and reproductive investment are strongly linked to rainfall at origin (Berger and Ludwig, 2014; Berger et al., 2017; Berger J. D. et al., 2020). Despite the importance of phenology in ecotypic adaptation of wild lupins, there is very little variation in vernalization response (Taylor et al., 2019). Similarly, most wild *Cicer* species also appear to be consistently vernalization responsive (Berger et al., 2005), suggesting that other regulatory mechanisms such as photoperiod or temperature responses or earliness *per se* are responsible for the phenological differences observed across wild germplasm collections. Indeed, wild *Cicer* species appear to be extremely photoperiod responsive (Sharma and Upadhyaya, 2019) and investigations are ongoing to reveal interactions between all three phenology regulators (vernalization, photoperiod, temperature) in order to understand the regulation of flowering time and what CWRs might bring to the crop.

Conversely, downregulation of the vernalization response has played a key role in the domestication of chickpea and both lupin species as these crops moved away from their cool-cold winter Mediterranean origins. Selection for earliness and high early vigor were key breeding criteria in both lupin species in their domestication as spring-sown crops because timely maturity in the cooling late central-eastern European summer was essential to reliable seed production (Hondelmann, 1984). When the crops moved to relatively warm-winter Mediterranean climates in Australia, where vernalization is a mostly unreliable regulator of phenology, it was necessary to identify unresponsive mutants to start the industry effectively (Gladstones and Hill, 1969). Now, both Australian and European Lupin breeding is dominated by the vernalization unresponsive *FT* alleles (Taylor et al., 2019, 2021). In chickpea vernalization was presumably downregulated in the Bronze Age introduction of the crop to South Asia and its hypothesized return as a Mediterranean spring-sown crop (Abbo et al., 2002, 2003; Redden and Berger, 2007). Unlike lupin, the much older chickpea crop has formed distinct ecotypes with different flowering regulatory mechanisms appropriate to each region (Berger et al., 2011). Thus, chickpea landraces become increasingly temperature responsive from the Mediterranean through north, central and southern India, temperature response being strongly correlated to collection site temperature during the vegetative phase (Berger et al., 2011). Among Eastern Mediterranean germplasm photoperiod and temperature response were negatively correlated, a relationship that is likely to have been essential in the colonization of warmer climates in South Asia where a strong photoperiod responsive is maladaptive, particularly in Southern India where chickpea flowers under declining day length.

Divergence in adaptive strategies between crops and wild relatives provides opportunities to modify crop adaptation to new and marginal environments, while understanding which traits are selected for what regions adds useful context to the problem. For example, genes from chickpea wild relatives would be invaluable in the creation of a winter cultivar because these appear to have been lost in evolution of the crop. Indeed, chickpea wild relatives are more winter hardy than domesticated chickpea (Singh et al., 1990, 1994) and appear to have greater reproductive chilling tolerance as well (Berger et al., 2012b). Efforts are currently underway to improve cold tolerance in chickpea using wild relatives. In narrow-leafed lupin the strong selection for earliness has created a bottleneck which is limiting yield potential in higher rainfall environments where longer season cultivars will be better adapted (Berger et al., 2012a; Chen et al., 2017). As a result, considerable effort is being invested in the search for other regulatory levers with which to delay flowering in lupin without re-introducing an obligatory vernalization requirement (Taylor et al., 2019, 2021). Wild germplasm is playing an important role in this. These examples stand out because phenology has played such a central role in the divergent evolution of crop and wild relative in both chickpea and lupin.

When the differences between crop and wild relative are more obscure, it becomes more difficult to generalize about the adaptive potential that may be exploited from CWR. Drought stress often occurs in marginal environments as described earlier. Among annual plants the principal adaptation strategy is stress avoidance through appropriate phenology, with some very limited capacity for drought postponement through water acquisition (e.g., deep roots) and conservative water use (e.g., stomatal closure), and very short-term tolerance through osmotic adjustment (Ludlow and Muchow, 1990; Berger et al., 2016). Given the widespread selection for earliness in domesticated crops compared to their wild relatives, it is difficult to argue that CWR will improve the capacity for drought avoidance in our crops. However, CWR may be a useful source of drought postponement or tolerance traits, given that these are likely to be under selection in wild populations where individuals compete amongst themselves and often also have long, indeterminate lifecycles which include periods of transient drought stress (Berger et al., 2016). Lysimetry has demonstrated that the wild *Cicer* species, *C. echinospermum* and *C. reticulatum* extract more water both under terminal drought and well-watered conditions than does domestic chickpea (Berger J. et al., 2020). At this stage the underlying mechanism is unknown. If the greater water use extraction of wild *Cicer* is explained by greater vegetative investment and associated lower water-use efficiency, it will be maladaptive in the chickpea crop; if it represents a capacity to tolerate lower water potential, however, it may be useful. Wild *L. luteus* has a greater capacity to maintain leaf water content at low water potential than the domesticated crop, probably through osmotic adjustment (Berger and Ludwig, 2014; Berger J. D. et al., 2020). Surprisingly this capacity was only found in high, rather than low rainfall ecotypes and was suggested as a bet-hedging strategy to postpone self-induced transient drought caused by very high water-use of high rainfall ecotypes. Currently, there are not enough ecophysiological studies of annual adaptive strategies over contrasting climates to generalize about the likelihood of finding these sorts of adaptive traits among CWR. There has been no evidence for similar drought stress tolerance on low and high rainfall ecotypes of wild *L. angustifolius* (Berger J. D. et al., 2020), Conversely Yousfi et al. (2010) detected greater osmotic adjustment capacity in low compared to high rainfall ecotypes of *Medicago truncatula* and *M. laciniata*. We hope that future germplasm screening efforts used in crop improvement will address this shortcoming by studying material from contrasting, well characterized collection sites.

## Desirable Crop Wild Relatives Phenotypic Traits to Improve Crop Adaptation to Marginal Environments

Selection attributes according to the crop objective are related to classical domestication traits, such as reduced seed and fruit dispersal, changes in plant structure, plant phenology, seed dormancy, palatability, or acquisition of modified fruit size and shape in vegetable crops (Alonso-Blanco et al., 2009; Sakuma et al., 2011; Meyer et al., 2012; Zohary et al., 2012; Abbo et al., 2014; Fuller and Allaby, 2018; Ogutcen et al., 2018; Smýkal et al., 2018; Iqbal et al., 2020). Much progress has been made in the most widely grown and intensively selected crops (rice, wheat, soybean, sugarcane, tomato and potato), but improvement using CWR is more recently (Hajjar and Hodgkin, 2007; Shelef et al., 2017; Zhang et al., 2018; Zsögön et al., 2018; Fernie and Yan, 2019). CWRs provide unique reservoirs of genetic diversity for crop improvement (Cowling et al., 2009; Redden, 2013; Matesanz et al., 2020; Bohra et al., 2021). They have typically been used in breeding to introgress a small number of major genes (for insect/disease-resistant varieties), in contrast to adaptation that is controlled by many small-effect quantitative trait loci and complex interactions which are more difficult to harness (Hajjar and Hodgkin, 2007; Zhang et al., 2018; Zsögön et al., 2018). The introgression of adaptive diversity from CWR to domesticated crops provides breeders with new tools for crop improvement (Smýkal et al., 2018). As environmental changes associated with domestication should favor resource-acquisition strategies compared with the resource-conservation strategies of wild relatives (Redden, 2013; von Wettberg et al., 2014; Vilela and González-Paleo, 2015), breeders are faced with the challenge of a trade-off between productivity and adaptation. The development of a variety with high adaptation (survival) under a marginal environment, but very low productivity would not be desirable (Richards, 2006; Berger et al., 2016).

Cultivars with tolerance to drought, heat, salinity, soils with extreme pH (alkaline or acidic) and low fertility, as well as various biotic factors are known to be required in marginal environments (Redden, 2013; Quezada-Martinez et al., 2021). Irrespective of the pre-breeding technique it is important to understand the phenotypic traits that confer adaptation for identification in any given marginal environment and for agronomic management (Wilke and Snapp, 2008). Appropriate phenotypic traits (morphological, anatomical, and phenological) that are easily distinguishable and inexpensive to measure in the field, suitable for pre-breeding programs in developing countries, where marginal environments predominate, could be considered. However, the breeding efficiency might be low (Araus and Kefauver, 2018). On the contrary, adaptation to salinity through the evaluation of osmoprotectants, reactive oxygen scavengers (ROS), stress proteins and ion/proton transporters, or the resistance to diseases-pests not strongly associated with specific phenotypic traits, requires more complex screening and modern techniques (Araújo et al., 2015). Since many CWR can help stabilize and increase productivity in marginal agroecosystems, the phenotypic traits that provide these and other types of services of candidates for domestication should be described. Table 1 presents some phenotypic traits that are related to the plant responses to marginal environmental factors. These traits can guide breeders who are seeking new germplasm for domestication.

**TABLE 1:** Linking phenotypic traits and adaptation strategies that could be used as pre-breeding criteria in a population improvement program in grain and pasture crops.

Specific adaptation strategy	Phenotypic trait of interest	Target environment	References
Drought escape.	High seed size. Rapid germination and growth rates (high seedling vigor).		Thomson and Siddique, 1997; Berger et al., 2002, 2016; Richards, 2006; Reynolds et al., 2007; Sadras and Richards, 2014; Araújo et al., 2015; Duc et al., 2015; Gardarin et al., 2016
*The life cycle is completed before a severe water deficit develops.*	Early flowering and physiological maturity phenology. Determinate growth habit. High specific leaf area (SLA). Stay-green. Dry matter remobilization and high reproductive investment (harvest index). Lengthened seed-filling period.	Terminal drought (or *end-of-season drought*)	
Drought avoidance/postponement.	Phenology adjustment (*ability to modify growth and flowering phases according to occurrence of rainfall events*). Root:shoot ratio increase.	Transient stress (or *droughts occurring at any time of the growing season*)	Berger et al., 2002, 2016; Richards, 2006; Reynolds et al., 2007; Wilke and Snapp, 2008; Fernández et al., 2012
*When maximizing water uptake and/or reduced soil water depletion.*	Leaf area reduction. Leaf thickness increase. SLA* reduction. Leaf senescence delay. Leaf movement (paraheliotropic, rolling). Leaf (canopy) temperature reduction.		
Drought and temperature stress tolerance. *Specific adaptation to water deficit through organs or processes.*	Dense leaf pubescence. Smaller and thicker leaf. High thick leaf cuticle. High leaf wax. Low SLA*. Deep root. High root:shoot ratio. Green photosynthetic organs other than leaf (stems on shrubs, beard in cereals). Nyctinastic leaf movements.	Transient and terminal drought	Sandquist and Ehleringer, 1997; Passioura, 2006; Richards, 2006; Reynolds et al., 2007; De Andrés et al., 2008; Wilke and Snapp, 2008; Araújo et al., 2015; Vilela and González-Paleo, 2015; De la Rosa et al., 2020
	Leaves highly reflective or orientated at steep angles.		
Weed competitiveness	Low seed dormancy Rapid growth rates. High plant height. High specific leaf area. Dense leaf canopies. Spread root systems. High tillers/stems per plant. High dry matter.	Mild, transient stress	Hoad et al., 2006; Duc et al., 2015; Gardarin et al., 2016
Winter survival	Survival testing (at < 0°C) Median lethal temperature (LT_50_) Regrowth ability after freezing Pubescence	Freezing winter	Badaruddin and Meyer, 2001; Brandsæter et al., 2002; Wilke and Snapp, 2008; Araújo et al., 2015; Wiering et al., 2018
Prevents germination in unfavorable conditions	Seed dormancy High seedling growth rate	Initial drought	Loi et al., 2005; Norman et al., 2005; Gardarin et al., 2016
Ability to recover from transient damage (grazing, predation and biotic stress)	Hypogeal emergence. Phenology plasticity (*to extend the growing season*). Secondary stem/shoot production. Indeterminate growth habit. High allocation to roots or storage organs. Low HI or RE (reproductive effort). Deep-rooting.	Grassland – Pasture crop	Wilke and Snapp, 2008; Annicchiarico et al., 2015; Araújo et al., 2015; Duc et al., 2015; Renzi, 2020

*Specific leaf area (SLA) = leaf surface area (cm^2^)/leaf dry mass (g).

**FIGURE 2:**
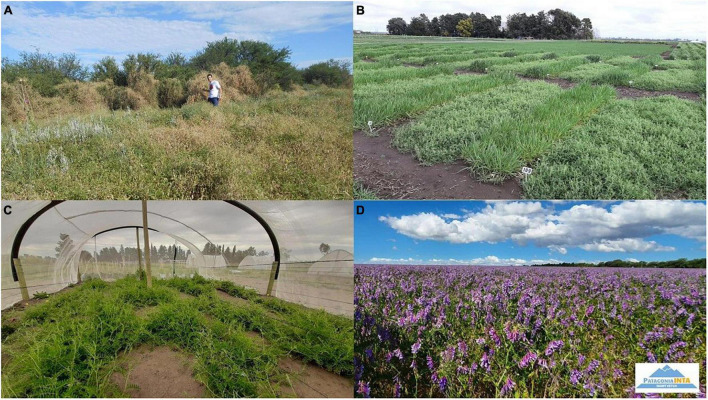
Seed collection **(A)**, plant breeding **(B)**, multiplication **(C)**, and agronomic use **(D)** of the improved cultivar of hairy vetch (*Vicia villosa*) cv. Patagonia INTA as forage and cover crop.

Note that some traits are not directly related to yield, or even appear to be indirectly associated, but will favor adaptation in a marginal environment in the long term (Duc et al., 2015; Vilela and González-Paleo, 2015). For example, it is known that the use of high reproductive biomass (high harvest index) as a key selection criterion leads to increased wheat yield (Reynolds et al., 2007). However, in marginal environments in the northern Patagonian region of Argentina, it may be preferable to select high plant height and late-cycle wheat to cope with transient stress while maintaining the yield ceiling (∼ 1–1.5 t ha^–1^). Tall crops could favor competition with weeds, and the late-season genotype could prolong the duration of root growth, allowing the utilization of water and nutrients deeper in the soil profile (Richards, 2006). In addition, higher post-harvest residues with firmer stubble could increase soil cover, favoring soil moisture retention and avoid erosion processes that frequently occur in bare soils (Silenzi et al., 2012). Often remnant vegetative biomass is used as forage or for fuel. This is one of the reasons why traditional local varieties are still used instead of modern ones in this marginal environment (Cantamutto et al., 2016). In the case of forage legumes, the simple selection of a major trait (biomass) with four adaptive traits was useful for improving a semi-domesticated species such as hairy vetch (*Vicia villosa*) (Figure 2). The adaptive traits were late phenology, aboveground morphology (high plant branching), dense leaf pubescence and high seed dormancy. The long period of vegetative production was associated with the ability to modify the growth and flowering phases according to the occurrence of rainfall events. Successful adoption by farmers has occurred with the cultivar Patagonia INTA, with a high biomass and regrowth capacity after grazing, tolerance to transient drought, resistance to cold, and adequate natural reseeding (Renzi, 2020). The lesson is that adaptive traits have to be considered with the target environment *and* end user in mind; seed yield may not always be the dominant criterion.

A less common but promising approach is the domestication of perennial grain crops. Traditional systems relying on annual crops have substantial negative impacts on ecosystem functions (i.e., nutrient cycling, water quality and carbon emissions, salinity, soil erosion and degradation) (Tilman et al., 2011). These problems can be reduced through the introduction of perennial crops in some environments (González-Paleo and Ravetta, 2015). This is illustrated by perennial Silphium oilseed (Heliantheae), with ideotypes with desirable traits for improving long-term yields and ecosystem services (Runck et al., 2014; Van Tassel et al., 2017). Kernza, a large-grained variety of intermediate wheatgrass (*Thinopyrum intermedium*) (DeHaan et al., 2020), is similar. The introduction of greater diversity in cropping systems through biotypes for mixed cultivation (“ideomixes”), with high resource-use complementarity, is also used as a novel approach by breeders (Litrico and Violle, 2015). For forage purposes, the domestication of shrub-tree legumes for livestock production has the potential not only to enhance productivity, but it could also provide multiple environmental benefits, such as providing shade for cattle, restoring degraded lands and mitigating greenhouse gas (GHG) emissions (Muir et al., 2018). The concept of the traditional ideotype in marginal environments needs to be discussed, and the ecosystem services of these should be considered (Duc et al., 2015). While introduction of perennial crops may not suit every marginal environment (such as those that experience extended hot and dry summer seasons), more research effort should be directed toward exploring the potential fit of perennial crops to different marginal environments.

Different drought scenarios (opportunity, extent, intensity and frequency of drought) and heat-stress are the most common and important stress factors in marginal lands (Chapman et al., 2000; Reynolds et al., 2007; Berger et al., 2016; Sehgal et al., 2018). This combination of changes in temperature and rainfall patterns is driving the development of drought-adapted crops. Table 1 details the different adaptive strategies to drought and the associated phenotypic traits; however, the integration of traits into an adaptive strategy that explains how species or genotypes address drought stress should be analyzed in a specific context that considers environmental selection pressures.

The phenotypic traits between drought postponement and tolerance may be overlapping in the same directions (co-gradient variation). Plant adjustments under stress conditions (plasticity) as opposed to tolerance as a constitutive trait of the species independent of water and/or heat stress are considered. Both the genetic differentiation in phenotypic traits and the plasticity of those traits are complementary mechanisms contributing to plant adaptation to environmental resource fluctuation (Matesanz et al., 2020). Uniform-rich habitats or consistently stressful environments (homogeneous and predictable low-resource environments) promote genetic differentiation rather than plasticity. On the contrary, unpredictable environments can lead to increased phenotypic plasticity (Vilela and González-Paleo, 2015; Espeland et al., 2018). Thus, the plasticity could represent most of the phenotype variation in some traits (phenology, leaf morphology) when compared with the genetic contribution; it has been suggested that it could be beneficial under climate change scenarios (Nicotra et al., 2010). In order to detect “plastic ideotypes” and exploit the plasticity in pre-breeding programs, a wide range of environments in order to fit models of phenotype versus environment should be included (de Felipe and Alvarez Prado, 2021).

In this context, CWR show high genetic variation for adaptation and may represent potential candidates for introduction into marginal areas. In addition to adaptation to different stress conditions, the ability to fix atmospheric nitrogen (N) is important in legumes and should be considered (Kebede, 2021). The percentage of nodulated plants and the number of nodules per plant are associated with N fixation in some species (Provorov and Tikhonovich, 2003). For pre-breeding purposes, the qualitative plant vigor rating, biomass, and the N measurements from non-destructive samples collected during flowering or maturity can be used (Muller et al., 2021). As the plant-associated microbes are closely linked, abiotic stresses can affect either the host plant, the *Rhizobium*, or the *Rhizobium-*legume symbioses, decreasing the N-fixing activity (Zahran, 1999). Marginal environments harbor and select diverse microbial communities, and the isolation of effective rhizobia from wild legumes for inoculating other legume crops could be an effective strategy (Ayangbenro and Babalola, 2020). Screening for tolerant N-fixing bacteria strains, as in other growth-promoting (PGP) organisms, should be considered in the selection of plant species/cultivar-specific microbial populations (Coba de la Peña and Pueyo, 2011).

Stress tolerant wild plants, such as halophytes, secretohalophytes, xerophytes and thermophytes, with a suite of desired features (food, economical or ecological) represent useful germplasm (Zhang et al., 2018), and the ecological niche models can be used to predict the adaptation in a target marginal environment (Phillips et al., 2006; Redden, 2013). Understanding the adaptive strategy simplifies crop improvement by allowing breeders to introgress new traits through traditional breeding methods or modern selection techniques (Berger et al., 2016). Likewise, knowledge of phenotypic traits in wild species would estimate the probability of adaptation *a priori*, and the global plant functional trait databases may be used as a screening tool (Kattge et al., 2011; Saatkamp et al., 2019).

## The Potential Use of Demographic Modeling for Crop Wild Relatives With High Non-Domestication Traits, Such as Low-Cost Management

Domestication could result in the loss of potentially useful “wild traits” that confer biotic and abiotic stress tolerance or specialized resource-use strategies (Van Tassel et al., 2017; Nagel et al., 2019). Pizza et al. (2021) showed that in *Clarkia pulchella*, after the eighth generation of farm cultivation, the relative fitness of the wild plants was significantly greater than the farmed plants, especially under drought stress. Genetic diversity lost over generations may be mitigated by growing out a large number of unrelated individuals (Basey et al., 2015). On the other hand, obtaining an ideal cultivar for a marginal environment can take many years of breeding resulting in abandoned programs due to low financial resources (Hajjar and Hodgkin, 2007; Muir et al., 2014; Fernie and Yan, 2019). Demographic modeling, especially in adapted pasture biotypes due to the predominance of livestock systems in marginal environments, can be used to advance their use in real production systems, until getting an advanced cultivar.

**FIGURE 3:**
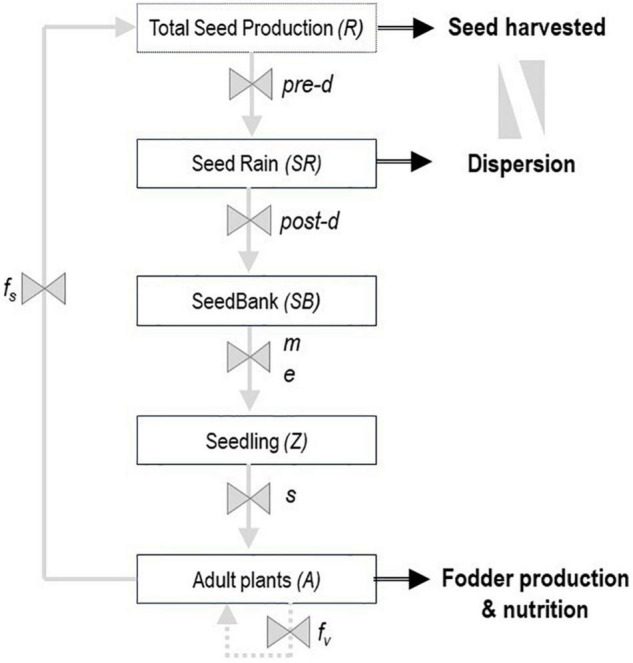
Conceptual model of the life cycle of an annual or perennial pasture biotype. Boxes indicate state in the plant life cycle. Gray arrows indicate the processes, and black arrows the agronomic traits of interest for pre-breeding. Process variables: pre-d, predation pre-dispersal; post-d, predation post-dispersal and seed rain losses; m, mortality of seeds in the soil; e, germination and seedling emergence; s, seedlings survivorship; fs, seed fecundity; and fv, vegetative reproduction.

Adapted biotypes, which are not necessarily domesticated, can be very difficult to cultivate. Poor agronomic performance introduces numerous practical issues for breeders, such as difficulty in recovering a sufficient number of seeds and not being able to use standard equipment (Nutt and Loi, 1999; Nichols et al., 2012). For fodder production or restoration purposes, these traits can be used for the dispersal of desirable biotypes through demographic modeling. Dynamic population models have proven to be very useful for understanding survival and dispersal strategies of wild and semi-domesticated species. This approach is widely documented for reducing survival in weed species under different production systems (González-Andujar and Fernández-Quintanilla, 1993, 2004; Forcella et al., 2000; Colbach et al., 2005; Holst et al., 2007; Gardarin et al., 2012), as well as promoting it in legume and grass pastures (Taylor et al., 1991; Komatsuzaki, 2007; Thapa et al., 2011; Renzi et al., 2017). These models, in their simplest form, consist of five stages from seed production, seed dispersal and input into soil seed banks, through germination and seedling emergence to the reproductive phase (adult plants) (Figure 3). The study of these stages and processes can be the basis for identifying the causes of failure or success in the establishment and performance of the selected biotypes (Renzi et al., 2019).

The first requirement for this approach is to find the biotype(s) adapted to the target marginal environments, with acceptable forage yield and nutrition. Secondly, to reach a successful commercial scale, it is important to have seed available for farmers (Runck et al., 2020). To improve the harvestable seed yield, less seed shedding after physiological maturity, is a key objective for pre-breeding programs (Nichols et al., 2012). When the number of hectares that can be planted for cultivation I from a single hectare of seed production (S) is lower than 20 (C/S < 20), models that assist natural reseeding could be a good option. This is the case of *Vicia villosa* which is well-adapted to semi-arid temperate environments, but incompletely domesticated due to extreme indeterminate growth, non-uniform maturity, large seed losses due to uneven pod dehiscence, and seed dormancy (Kissing Kucek et al., 2020a,b; Renzi et al., 2020). The demographic model was useful for expanding their adoption by farmers in marginal lands in Argentina (Renzi et al., 2017, 2019). Several options of legumes with self-regeneration potential have been published (Carr et al., 2005; Loi et al., 2005, 2012; Norman et al., 2005; Ovalle et al., 2005; Driouech et al., 2008; Walsh et al., 2013). In a scenario of very low seed production, with a C/S ratio < 10, the natural seed dispersal should be considered in addition to the field emergence model. Under this premise, planting in patches or stripes (<<seed doses per ha) in function of the dispersal ability together with appropriate management will reduce the need for high initial seed availability. This management could be carried out in intercropping using biotypes with high resource-use complementarity (Litrico and Violle, 2015; Mazzafera et al., 2021). Thus, estimates of plant dispersal distances could be useful not only for ecological studies, but also for agronomic purposes. The dispersal traits of tall perennial pasture grasses suggest the potential applicability of this modeling approach (Thomson et al., 2011; Bullock et al., 2017). Nevertheless, complementary studies of temporal and spatial demographic dynamics are necessary.

## Pre-Breeding Techniques for Transferring Useful Traits to the Crop

Crop wild relatives are an untapped source of genetic diversity and a critical resource to meet food security needs and the challenges of new production systems, especially in response to climate change (Zhang et al., 2017; Bohra et al., 2021). However, three major factors discourage the use of CWRs in plant breeding (Zamir, 2001): (1) poor agronomic performance of CWRs, mostly due to the presence of undesirable traits such as shattering and seed dormancy; (2) crossing barriers between crops and their wild relatives, which limits the production of fertile offspring; and (3) the transfer of undesirable traits physically linked to the target trait, known as linkage drag (Zamir, 2001; Hajjar and Hodgkin, 2007; Shelef et al., 2017; Fernie and Yan, 2019). Therefore, to facilitate the use of CWRs in breeding programs, it is necessary to focus on the development of systematic strategies for the characterization and use of CWRs. Pre-breeding here refers to all activities designed to identify and transfer desirable traits or alleles from genetic materials that cannot be used directly in plant breeding (Prohens et al., 2017; Tefera, 2021). Pre-breeding produces intermediate genetic materials that can be used for breeders to produce elite cultivars. This section provides a review of distinct pre-breeding strategies to facilitate the use of CWRs in breeding programs, focused on the utilization of CWR for improving the tolerance to stressful conditions associated to marginal agricultural lands.

In the broad sense, strategies to incorporate CWR into breeding programs can be classified as **“choose first”** or **“cross first”** (Dempewolf et al., 2017). In “choose first” wild accessions are chosen from a larger number of accessions, based on phenotypic, genotypic, or eco-geographic merits. Chosen accessions are expected to carry a desirable trait, then they are used in a target crossing design. In “cross first” one or more wild accessions are crossed with elite cultivars, and then the progeny is evaluated, this approach is useful when the trait of interest cannot be directly measured in the wild donor.

### Choose First Strategy: Target Hybridization

Target hybridization is the classic example of the “choose first” strategy. Generally, a single wild accession carrying a single trait of interest (e.g., herbicide resistance, disease resistance) is used as donor and one elite cultivar, with good agronomic performance but lacking the trait of interest, is used as recurrent parent (Breseghello and Coelho, 2013). Crossing and backcrossing with positive selection for the target trait are used to incorporate the target trait and to recover most of the recurrent parent genome. This approach is routinely used in breeding programs to improve tolerance to pests and diseases (Hajjar and Hodgkin, 2007; Larkan et al., 2013; Seiler et al., 2017; Bohra et al., 2021). Using this approach, molecular markers can be used to map the region underlying the trait of interest, and to accelerate the recovery of the elite genome through assisted backcrossing (Breseghello and Coelho, 2013). The first step of target hybridization is to identify the suitable wild accession carrying the trait of interest (Zamir, 2001; Bahrami et al., 2021). As mentioned above, wild accessions can be selected based on phenotypic, genotypic, or eco-geographic merits. For phenotypic selection, several wild accessions are directly screened for the trait of interest along with elite cultivars; accessions outperforming elite cultivars for the target trait are selected for the next stage (Bahrami et al., 2021). Phenotypic screening has been used to identify wild accessions with increased tolerance to several stressful conditions associated to marginal agricultural lands, such as salinity in wild tomato (Pailles et al., 2020), freezing in wild sunflower (Hernández et al., 2020), drought in wheat and barley (Nevo and Chen, 2010), heat in wild rice (Scafaro et al., 2016; Bheemanahalli et al., 2017), wild sunflower (Hernández et al., 2018, 2020), and wild barley (Bahrami et al., 2021). Genotypic information can also be used to identify valuable wild accessions. For example, Buitrago-Bitar et al. (2021) identified wild accessions of tepary bean carrying novel alleles for previously identified drought-responsive genes. With the increasing amount of reported candidate genes for abiotic stress tolerance, genes-specific hybridization probes can be designed to target multiple candidate genes at once in many samples before next generation sequencing (Weitemier et al., 2014), thus accelerating the discovery of novel alleles of relevant genes. Lastly, eco-geographic tools can be used to identify possible sources of abiotic stress tolerance (e.g., Khazaei et al., 2013; Kantar et al., 2015; Stenberg and Ortiz, 2021). Eco-geographic approaches gather environmental variables from collection sites such as temperature, precipitation, and soil characteristics. This approach assumes that wild accessions from sites prone to a given abiotic stress (e.g., drought, cold or heat) likely carry adaptive alleles to cope with these environmental challenges (Kantar et al., 2015; Anderson et al., 2016). Phenotypic, genotypic, and eco-geographic approaches can be combined. Landscape genomics is a powerful approach that combines eco-geographic and genomic data to identify loci underlying environmental adaptation (Joost et al., 2007). This approach does not require phenotypic experiments, thus allowing the evaluation *in silico* of hundreds to thousands of accessions. Several climatic databased, including WorldClim (Fick and Hijmans, 2017), Envirem (Title and Bemmels, 2018), and SoilTemp (Lembrechts et al., 2021), along with genomic information can be used to link geographic coordinates of any accession with local environmental conditions and genomic data, increasing the value of *ex situ* collections. Recent examples focused on wild relatives of soybean (Anderson et al., 2016), wheat (Brunazzi et al., 2018), narrow-leafed lupin (Mousavi-Derazmahalleh et al., 2018), and chickpea (von Wettberg et al., 2018). Once the suitable accession is identified, a crossing and backcrossing design with an elite cultivar is initiated. As this approach uses only one wild accession as donor, genome-wide increases of genetic diversity are not expected. The advantage of this approach is the efficient transfer of traits regulated by a few genes, such as disease resistance (Seiler et al., 2017), and it is well-known by breeders. However, it is time-consuming and its success in traits with complex genetic architecture is not guaranteed.

### Cross First Strategy: Wide Hybridization

The cross first strategy consists of developing populations of lines with introgressions from CWR into the genetic background of crops, aiming to generate elite materials carrying genome fragments from CWR (Tanksley and McCouch, 1997; Dempewolf et al., 2017; Prohens et al., 2017). Such populations can be used to identify genomic regions underlying adaptive traits, but they can also be directly incorporated into breeding programs (von Wettberg et al., 2018; El Haddad et al., 2021). There are many ways for developing populations with introgression from CWR; Chromosome Segment Substitution Lines (CSSL), Nested Association Mapping (NAM) populations, and Multiparent Advanced Generation Intercross (MAGIC) populations. These populations are often developed in an unfocused manner regarding target traits, i.e., the aim is to generate populations with as high genetic diversity from CWR as possible, thus populations are useful to map most agronomic traits in many environments. However, multi parent populations can be developed in a focused manner, selecting parents with particular features, such as tolerance to biotic or abiotic stress (reviewed in Prohens et al., 2017; Scott et al., 2020).

### Chromosome Segment Substitution Lines

Chromosome Segment Substitution Lines were designed and used for mapping QTL in many crops (Balakrishnan et al., 2019). CSSL form a population of lines with chromosomal segments from one wild accession into an elite background (Balakrishnan et al., 2019). Ideally, each CSSL harbors a single segment of the donor and the whole donor genome is distributed segment-wise in the entire population of CSSL. In pre-breeding, CSSL has been used in several crops, such as rice, wheat, barley, maize, and soybean to identify and transfer alleles from wild species, including alleles associated to drought, heat, and freezing stress (reviewed in Balakrishnan et al., 2019). By definition, with this approach a single wild accession is used as donor and a single elite cultivar as recurrent parent, although CSSL populations using the same recurrent parent and distinct wild donors can be combined (Singh et al., 2020).

### Multiparent Populations

In the last decades, multiparent populations of crop species were developed and largely used to explore the genetic architecture of agronomic traits (Scott et al., 2020). Nested Association Mapping (NAM) and Multiparent Advanced Generation Intercross (MAGIC) populations are the most popular designs. They were designed to improve the resolution of QTL analysis with biparental populations and to reduce the confounding effects of population structure of association mapping (Gage et al., 2020). While NAM populations can be seen as a collection of biparental populations with a common parent (e.g., Yu et al., 2008; Hu et al., 2018), MAGIC populations have more complex crossing designs, commonly involving 4, 8, or 16 parents (Zamir, 2001; Gage et al., 2020; Scott et al., 2020). Such differences in the crossing designs determine that lines from NAM and MAGIC populations differ in their genetic composition, even when the same parents are used. In the NAM populations, lines are grouped into families, with as many families as donor parents, thus the genomes of NAM lines are a mosaic of only two parents, and haplotype diversity is constrained by the common parent (Zamir, 2001; Gage et al., 2020). On the contrary, the genomes of MAGIC lines are a mosaic of all founders, and haplotype diversity grows with the number of founders, although it is constrained by the number of lines in the population (Zamir, 2001; Scott et al., 2020). More than 50 multiparent populations were developed for several crop species (reviewed in Scott et al., 2020), however, only four of them included CWR as founders, three for barley and one for maize (Scott et al., 2020). In multiparent populations, the selection of founders is the most critical step, as it determines the pool of genetic variation segregating in the population. Choosing founders as genetically diverse as possible maximizes the population’s utility for mapping agronomic traits in multiple environments (Gage et al., 2020; Scott et al., 2020). However, for pre-breeding, a more conservative selection of founders will produce populations with higher overall agronomic performance, and therefore more attractive for breeders. In the case of NAM populations, after crossing the elite cultivar with CWR, one or more cycles of backcrosses can be implemented before selfing to improve the overall agronomic performance of the population (Chen et al., 2019). In the case of MAGIC populations, a trade-off between the overall agronomic performance and the novel genetic variation is expected, i.e., the larger the proportion of elite cultivars as founders the larger the overall agronomic performance and the lower the novel genetic variation, and vice versa.

### Genomic Selection and Multiparent Populations

The complex genetics controlling most abiotic stress tolerances presents a challenge for crop breeders to incorporate many CWR alleles into their breeding programs. Genomic selection (GS) is an efficient approach that was pioneered in animal breeding, where complex, polygenic traits are the norm (Goddard and Hayes, 2007). Genomic selection has been refined for plant breeding and adopted as a core methodology by many breeding programs (Crossa et al., 2017). This approach could be used to accumulate increasing numbers of alleles associated with stress tolerance and shift the breeding populations from stress-sensitive to stress-tolerant over many generations.

Genomic selection is increasingly common for a range of crops, e.g., barley (Gonzalez et al., 2021), pea (Al Bari et al., 2021), chickpea (Varshney et al., 2021), wheat (Muleta et al., 2017), and sorghum (Yu et al., 2016). However, GS to select within CWR collections has been proposed, but examples have yet to be published (Bohra et al., 2021). With the genotyping of larger CWR collections (e.g., Song et al., 2015; Sansaloni et al., 2020), it is expected this approach may be used in the future as it has been proven effective in plant breeding programs (Merrick and Carter, 2021). The importance of GS may increase for CWR pre-breeding programs as it allows for efficient introgression of beneficial alleles for polygenic traits with reduced linkage drag (Wang et al., 2017). Genetic selection has the advantage of selecting superior progeny based on genotypes alone using phenotyped training populations (Meuwissen et al., 2001). The first demonstration of GS in pre-breeding was in exotic maize populations successfully selecting for yield (Combs and Bernardo, 2013). Genomic selection in CWR pre-breeding was also validated recently in soybean (Beche et al., 2021). Very good prediction accuracies for several quantitative traits were reported using a NAM breeding population with three wild Plant Inventory parents (Beche et al., 2021). An excellent example for pre-breeding for marginal lands using focused identification of germplasm strategy (FIGS) is found in studies with barley, wheat and faba bean landraces (Endresen et al., 2011; Khazaei et al., 2013). FIGS is another machine learning application that takes multivariate data sets of *a priori* knowledge (phenotypes, environmental) to identify the potential most useful accessions (Bari et al., 2012). Stenberg and Ortiz (2021) suggest improving the FIGS model of adaptive processes (natural selection) by adding non-adaptive processes (gene flow and genetic drift) to hit the most intensive evolutionary hotspots to capture elusive traits followed by genomic selection.

In summary, the “choose first” strategy is more suitable for incorporating traits regulated by one or a few major loci (e.g., herbicide or disease resistance alleles) while the “cross first” is more suitable for polygenic traits, which is expected for most traits associated to adaptation to marginal agricultural lands. Multiple parent populations, especially MAGIC, are well-suited for the cross first strategy. Such populations are useful to discover agronomically relevant loci, to improve genetic diversity of pre-bred populations, and to directly incorporate them to breeding schemes. However, we identified a lack of multiparent populations (MPPs) that include CWR as founders, meaning that current MPPs are exploiting only a small fraction of species’ genetic diversity. Therefore, we call for a systematic development of MAGIC populations, which should include CWR, elite cultivars, and landraces as founders. The outlined advantages of MAGIC populations for both basic science and plant breeding should facilitate the creation of public-private pre-breeding partnership programs aiming to develop cultivars well-adapted to marginal agricultural lands. Although universal MAGIC populations can be used to discover agronomically relevant loci by multiple research groups, we encourage (when possible) the development of MAGIC populations with local founders to exploit local adaptation.

## *De novo* Domestication and Genome Editing

Since domestication began approximately 12,000 years ago, some significant events determined a deep change in the evolution of crops and their wild relatives. Fernie and Yan (2019) divided the history of crop improvement into four breeding generations, the first generation with breeding based on phenotype selection; the second generation with hybrid breeding and green revolution with dwarf plants and higher yields; a third generation called or “biotechnology-based breeding” with the arrival of genetically modified crops. Currently, the fourth generation of breeding is running, with genome editing and precision breeding. In fact, the fourth-generation breeding is determined by new plant breeding techniques like *cis*-genesis (Jacobsen and Schouten, 2007) and genome editing (Zsögön et al., 2017), mainly CRISPR/Cas (Sukegawa et al., 2021). *Cis*-genesis consists of the genetic transformation of a crop with CWR’s genes, without the introduction of reporters or selectable markers from other organisms, avoiding linkage drag. This is particularly interesting in the case of the secondary and tertiary genepool species (Harlan and de Wet, 1971) with strong hybridization barriers.

Genome editing techniques involve zinc-finger nucleases (ZFNs), transcription activator-like effector nucleases (TALENs), and clustered regularly interspaced short palindrome repeats associated protein 9 (Cas9), CRISPR/Cas systems (Manghwar et al., 2019; Sedeek et al., 2019; Van Tassel et al., 2020; Molla et al., 2021). ZFNs and TALENs are protein-based and require protein engineering for every user-defined sequence. Instead, CRISPR/Cas is a RNA-guided system that induces double-stranded DNA breaks by the action of Cas9 nuclease at a genome corresponding location (Belhaj et al., 2013; Altpeter et al., 2016; Rodríguez-Leal et al., 2017). Among genome editing techniques in plants, CRISPR/Cas9 has become the most popular (Altpeter et al., 2016; Pacher and Puchta, 2017; Das et al., 2019). The CRISPR/Cas9 system can generate stable and heritable mutations in genes that cannot be distinguished from a natural mutation at the same locus, without affecting the existing valuable traits (Belhaj et al., 2013; Arora and Narula, 2017). Since the main domestication genes in most of the major crops have been studied (see reviews by Kantar et al., 2017; Purugganan, 2019), the new gene editing tools would allow neo-domestication avoiding the linkage drag. *De novo* domestication or neo-domestication is defined as the targeted introduction of domestication genes into non-domesticated plants, this represents an important opportunity for fitting cultivated species to the climatic niche where they live (Fernie and Yan, 2019). In addition, genome editing allows to incorporate new adaptation attributes present in wild materials into cultivated ones, bringing resilience under the unfavorable conditions of climate change (Van Tassel et al., 2020). This is possible since the whole genome sequences for many crops and their wild species are now available (Bohra et al., 2021; Ramsay et al., 2021). However, it should be emphasized that this approach has yet to be fully tested and is unlikely to transfer all the beneficial traits accumulated in crops over centuries of generations, such as complex traits like grain yield, which is controlled by many genes.

Genome editing (GE) can be used for several purposes like to activate or suspend the function of any gene. Ghoshal et al. (2021) showed that with epigenome engineering it is possible to achieve heritable methylation, gene silencing, and delayed flowering time phenotypes. Hence, stable heritable epigenetic modifications in flowering time would allow researchers and breeders to overcome the crossing barriers of the secondary and tertiary genepool, facilitating crossings of CWRs with related crops (Cable et al., 2021). Genome editing can also be used to knock out genes. For example, increasing grain number and larger grain size in rice was possible by knocking out three mutations on negative regulators of yield (Mishra et al., 2018). In addition, knocking out the self-incompatibility gene S-Rnase allowed re-domestication of potato into an inbred-line based diploid crop for genetic improvement (Ye et al., 2018). This demonstrated the utility of this approach in four different Solanum tuberosum clones, opening possibilities in both future diploid breeding and basic research in self-incompatible crops.

Another application for GE is to generate novel alleles of any gene. For breeding drought tolerance in maize, novel allelic variation for ARGOS8 locus was generated by changing the DNA sequence at the native ARGOS8 (Shi et al., 2017). Genome editing could also add genes that do not exist in an original genome. There is also work on CRISPR/Cas9-mediated HDR (homology-directed repair) for editing genes that confer herbicide resistance, and to transfer an allele from a wild rice variant into a cultivated variant to increase yield potential. In addition, GE makes it possible to delete any sequence including large chromosomal fragments or even the entire chromosome (Xiao et al., 2013). This could be an advantage for the secondary and tertiary genepool species (Harlan and de Wet, 1971). Introgression of beneficial alleles from CWR into the cultivated gene pool has been a challenge when reproductive barriers are present. For instance, the genus *Helianthus*, native to North America, comprises 52 species including cultivated sunflower, *H. annuus* L. which has emerged as a model for genetic studies of adaptation, hybridization, and speciation. In particular, *H. annuus* and *H. petiolaris* are chromosomally divergent species, differing by a minimum of seven translocations and three inversions (Rieseberg et al., 1995). Notably, the first source of cytoplasmic male sterility for hybrid varieties’ development was discovered in *H. petiolaris* (Leclercq, 1969) and all currently grown sunflower hybrids are developed on cytoplasmic male sterility *petiolaris* type mothers. Chromosomal rearrangements are common in CWRs and drastically decrease recombination in crosses with crops by preventing the normal pairing of homologous chromosomes. The CRISPR/Cas system could be used to alter the frequency and guide homologous recombination to overcome these structural barriers between related species, thus opening new avenues to genomic restructuring and chromosomal engineering (Fauser et al., 2014).

The tertiary gene pool can also benefit from GE. Toda et al. (2019) demonstrated the possibility of genome editing in rice and maize zygotes, opening an avenue to gene manipulation in CWR wide crosses aiming to incorporate valuable traits to crops.

## Pre-Breeding Resources and Data Repository

Utilizing CWR in breeding and crop improvement requires that researchers have access to germplasm resources. International agreements, particularly the Nagoya Protocol to the Convention on Biological Diversity and the International Treaty on Plant Genetic Resources for Food and Agriculture, the two international agreements that govern the collection and sharing of crop genetic resources, enshrine access and benefit sharing as core principles, so that those who give germplasm resources benefit from them too. Since much CWR diversity comes from less developed regions of the world, yet has historically primarily been harnessed in the less diverse global north, there are real historical inequalities that access and benefit sharing (ABS) frameworks should address. Negotiations around ABS in treaties have increasingly focused on Digital Sequence Information (DSI) associated with CWR (Rohden and Scholz, 2022; von Wettberg and Khoury, 2022). Digital Sequence Information may encompass not only DNA sequence data associated with germplasm accessions, but also CWR passport data, traditional ecological knowledge on the preparation or use of varieties (Brink and van Hintum, 2022). This information, as our discussion above shows, can be incredibly powerful as a breeding tool, yet is very unequally distributed globally.

For those seeking germplasm access, there are a number of data sources for CWR as well as other databases with additional data and access for ordering seed or propagule samples. The Global Biodiversity Information Facility (GBIF) repository remains an essential database and data repository for discovery of species locations that includes most herbaria globally^1^ (Lane and Edwards, 2007). Efforts by the Global Crop Diversity Trust have greatly improved knowledge of target CWR taxa through their Genesys database^2^ (Dempewolf et al., 2014) and one of their partners with GRIN-Global^3^ (Postman et al., 2010). A number of CWR characterization, evaluation and genotype data sets can be found here under the Genesys ‘Dataset’ repository. Genesys and GRIN-Global are data repositories only; no user analysis tools are present or planned in this resource.

As noted by Brink and van Hintum (2022), most genebanks do not plan to host genotype data resources. However, the GRIN-Global development team now has modules to improve the storage and downloading of crop genetic resource genotype data sets (see footnote 3). Although sequence resources for CWRs are increasing, currently most plant genetic resource sequencing datasets are on domesticated material, notable examples are found for several crops (Song et al., 2015; Sansaloni et al., 2020; Hellwig et al., 2021). A very promising development is the expansion of the Germinate database to handle pre-breeding populations from CWRs (Raubach et al., 2021). The Germinate software will provide the CWR pre-breeding community critical tools for working with and selection/advancement of introgression lines for growing in marginal production areas. Adoption of FAIR data standards will increase the availability of CWR evaluation data for pre-breeding efforts (Wilkinson et al., 2016).

## *In situ* Conservation

For those harnessing CWRs, *ex situ* collections are critical as access points. But *ex situ* conservation alone is unable to fully conserve crop diversity, particularly for CWR that have historically been smaller parts of germplasm collections and harbor orders of magnitude more genetic diversity than domesticated accessions (Khoury et al., 2022). *In situ* conservation efforts for CWR have been growing over the last decade (Phillips et al., 2019) and can be essential to access traits of interest that are not covered by *ex situ* collections. *In situ* genetic reserve conservation may be defined as “the location, designation, management and monitoring of genetic diversity in natural wild populations within defined areas designated for active, long-term conservation” (Maxted et al., 1997). Some of the success in these efforts has been to harness existing conservation areas, often established for large animal preservation but also protecting CWR inadvertently. Dedicated efforts at *in situ* conservation have lagged, particularly in Vavilovian centers of origin where CWR taxa are most diverse and abundant. Protecting CWRs in their natural habitats is an essential strategy to prevent extinctions, especially where *ex situ* conservation of sufficient genetic diversity is practically or economically unfeasible (León-Lobos et al., 2012). There is a preference for *in situ* conservation, because it has the advantage of maintaining the dynamic evolution of the CWR diversity itself in relation to parallel biotic and abiotic changes. In the span of only three decades, for example, there was a shift in allelic diversity related to flowering time genes in wild barley (Qian et al., 2020) likely as response to global warming, and even an overwhelming local adaptation and ecological differentiation. Gap Analysis (e.g., Maxted et al., 2008) has been applied for hundreds of CWRs to find areas where *ex situ* collections and *in situ* conservation areas fail to protect the full range of diversity in CWR (Vincent et al., 2019; Khoury et al., 2020; Zair et al., 2021).

## Author Contributions

JR, JBr, CC, JBe, EW, MN, SU, FH, and PS contributed equally to writing and final editing. JR conceived the original idea. All authors contributed to the article and approved the submitted version.

## Conflict of Interest

The authors declare that the research was conducted in the absence of any commercial or financial relationships that could be construed as a potential conflict of interest.

## Publisher’s Note

All claims expressed in this article are solely those of the authors and do not necessarily represent those of their affiliated organizations, or those of the publisher, the editors and the reviewers. Any product that may be evaluated in this article, or claim that may be made by its manufacturer, is not guaranteed or endorsed by the publisher.

## Footnotes

1
www.gbif.org

2
www.genesys-pgr.org

3
www.grin-global.org
