# Corrigendum: How could the use of crop wild relatives in breeding increase the adaptation of crops to marginal environments?

**DOI:** 10.3389/fpls.2022.1101822

**Published:** 2022-12-02

**Authors:** Juan Pablo Renzi, Clarice J. Coyne, Jens Berger, Eric von Wettberg, Matthew Nelson, Soledad Ureta, Fernando Hernández, Petr Smýkal, Jan Brus

**Affiliations:** ^1^ Instituto Nacional de Tecnología Agropecuaria, Hilario Ascasubi, Argentina; ^2^ CERZOS, Departamento de Agronomía, Universidad Nacional del Sur (CONICET), Bahía Blanca, Argentina; ^3^ USDA Agricultural Research Service, Pullman, WA, United States; ^4^ Agriculture and Food, Commonwealth Scientific and Industrial Research Organisation, Wembley, WA, Australia; ^5^ Department of Plant and Soil Science, Gund Institute for Environment, University of Vermont, Burlington, VT, United States; ^6^ Department of Applied Mathematics, Peter the Great St. Petersburg Polytechnic University, Saint Petersburg, Russia; ^7^ The UWA Institute of Agriculture, University of Western Australia, Crawley, WA, Australia; ^8^ Department of Botany, Faculty of Science, Palacký University, Olomouc, Czechia; ^9^ Department of Geoinformatics, Faculty of Sciences, Palacký University, Olomouc, Czechia

**Keywords:** abiotic stress, adaptation, breeding, crop wild relatives, legumes, marginal environment

In the published article, there was an error in the Funding statement. The funding statement for the Key Development Project of the Department of Science and Technology was displayed as “PS was supported by the Grant Agency of the Czech Republic and Palacký University Grant Agency [IGA-2022_002]. JR work was supported by INTA PE-142 project. EW was supported by the USDA Hatch program through the Vermont State Agricultural Experimental Station.”. The correct statement is “PS was supported by the Grant Agency of the Czech Republic and Palacký University Grant Agency [IGA-2022_002]. JR work was supported by INTA PE-142 project. EW was supported by the USDA Hatch program through the Vermont State Agricultural Experimental Station. EW was also funded by the Ministry of Science and Higher Education of the Russian Federation as part of World-class Research Center program: Advanced Digital Technologies (contract No. 075-15-2022-311 dated 20.04.2022)”

The authors apologize for this error and state that this does not change the scientific conclusions of the article in any way. The original article has been updated.

## Publisher’s note

All claims expressed in this article are solely those of the authors and do not necessarily represent those of their affiliated organizations, or those of the publisher, the editors and the reviewers. Any product that may be evaluated in this article, or claim that may be made by its manufacturer, is not guaranteed or endorsed by the publisher.

